# Spatio-temporal heterogeneity of malaria morbidity in Ghana: Analysis of routine health facility data

**DOI:** 10.1371/journal.pone.0191707

**Published:** 2018-01-29

**Authors:** Timothy Awine, Keziah Malm, Nana Yaw Peprah, Sheetal P. Silal

**Affiliations:** 1 Modelling and Simulation Hub, Africa, Department of Statistical Sciences, University of Cape Town, Cape Town, South Africa; 2 South African Department of Science and Technology/National Research Foundation Centre of Excellence in Epidemiological Modelling and Analysis (SACEMA), University of Stellenbosch, Stellenbosch, South Africa; 3 National Malaria Control Program, Ministry of Health, Accra, Ghana; 4 Honorary Visiting Research Fellow in Tropical Disease Modelling, Nuffield Department of Medicine, University of Oxford, Oxford, United Kingdom; University of Minnesota, UNITED STATES

## Abstract

**Background:**

Malaria incidence is largely influenced by vector abundance. Among the many interconnected factors relating to malaria transmission, weather conditions such as rainfall and temperature are known to create suitable environmental conditions that sustain reproduction and propagation of anopheles mosquitoes and malaria parasites. In Ghana, climatic conditions vary across the country. Understanding the heterogeneity of malaria morbidity using data sourced from a recently setup data repository for routine health facility data could support planning.

**Methods:**

Monthly aggregated confirmed uncomplicated malaria cases from the District Health Information Management System and average monthly rainfall and temperature records obtained from the Ghana Meteorological Agency from 2008 to 2016 were analysed. Univariate time series models were fitted to the malaria, rainfall and temperature data series. After pre-whitening the morbidity data, cross correlation analyses were performed. Subsequently, transfer function models were developed for the relationship between malaria morbidity and rainfall and temperature.

**Results:**

Malaria morbidity patterns vary across zones. In the Guinea savannah, morbidity peaks once in the year and twice in both the Transitional forest and Coastal savannah, following similar patterns of rainfall at the zonal level. While the effects of rainfall on malaria morbidity are delayed by a month in the Guinea savannah and Transitional Forest zones those of temperature are delayed by two months in the Transitional forest zone. In the Coastal savannah however, incidence of malaria is significantly associated with two months lead in rainfall and temperature.

**Conclusion:**

Data captured on the District Health Information Management System has been used to demonstrate heterogeneity in the dynamics of malaria morbidity across the country. Timing of these variations could guide the deployment of interventions such as indoor residual spraying, Seasonal Malaria Chemoprevention or vaccines to optimise effectiveness on zonal basis.

## Background

Although significant progress has been made in studying the epidemiology of malaria in Ghana, investigating the relationship between malaria morbidity, across different ecological settings, using data from a single platform such as the District Health Information Management System (DHIMS), still remains largely unexplored.

Malaria is endemic in Ghana and varies across the country [1]. Ghana is characterised broadly by three ecological zones namely; the Guinea savannah zone which comprises the regions of northern Ghana, the Transitional forest zone located along the middle belt of the country and the Coastal savannah zone along the coast of the Atlantic Ocean [2–5]. Although the prevalence of malaria is observed all year around, it is seasonal with high incidence in the wet seasons [6].

In recent nationwide surveys, parasite prevalence among children 6–59 months old were observed to be 20.6% in 2016 [7]. There were however regional variations along the different ecological zones with relatively high parasite prevalence observed in the Transitional forest zone compared to other zones [7]. Malaria morbidity still accounts for about 40.0% of all out-patients attendance nationwide [8]. Although some successes have been made over the years following implementation of different policy interventions, much still remains to be achieved in the fight against malaria in Ghana [1].

Survival and prevalence of both the vector and parasite depend on various factors including rainfall and temperature which vary significantly across Ghana [9–11]. These meteorological variables have been shown to influence the transmission dynamics of malaria as they create a favourable environment needed to sustain vector replication, parasite development and human biting rates [12–14].

Understanding the diversity in the patterns and relationships between meteorological factors and incidence of malaria in Ghana, using readily available data on DHIMS, may be useful information upon which appropriate intervention assessment tools can be based. Although some studies have examined the relationship between rainfall, temperature and malaria morbidity in Ghana, they were all on small scale at the district population level [15–17]. This study therefore sought to explore and assess spatial and temporal heterogeneity of confirmed uncomplicated malaria cases captured at all public health facilities across the three different ecological settings in Ghana using time series analyses.

## Study design

Secondary data for uncomplicated malaria morbidity captured into a data cloud through DHIMS from 2008 to 2016 were analysed. Morbidity data were captured passively on daily basis in health facilities in all districts in Ghana. All patients presenting with suspected symptoms of malaria were tested by microscopy or Rapid Diagnostic Tests kits (RDT) before being diagnosed as a malaria case or not. In addition, meteorological data including monthly average rainfall and temperature from 2008 to 2016 obtained from regional weather stations were used.

## Study area and population

Ghana is located in the West African sub-region bordered by Togo to the east, Côte d’Ivoire to the west, Burkina Faso to the north and the Gulf of Guinea and Atlantic Ocean to the south. In 2016, the population was estimated to be 28 million people [18]. The country occupies a land size of about 238,540 km^2^ divided into ten administrative regions which are further divided into 237 districts and municipalities [1, 19].

**Fig 1:**
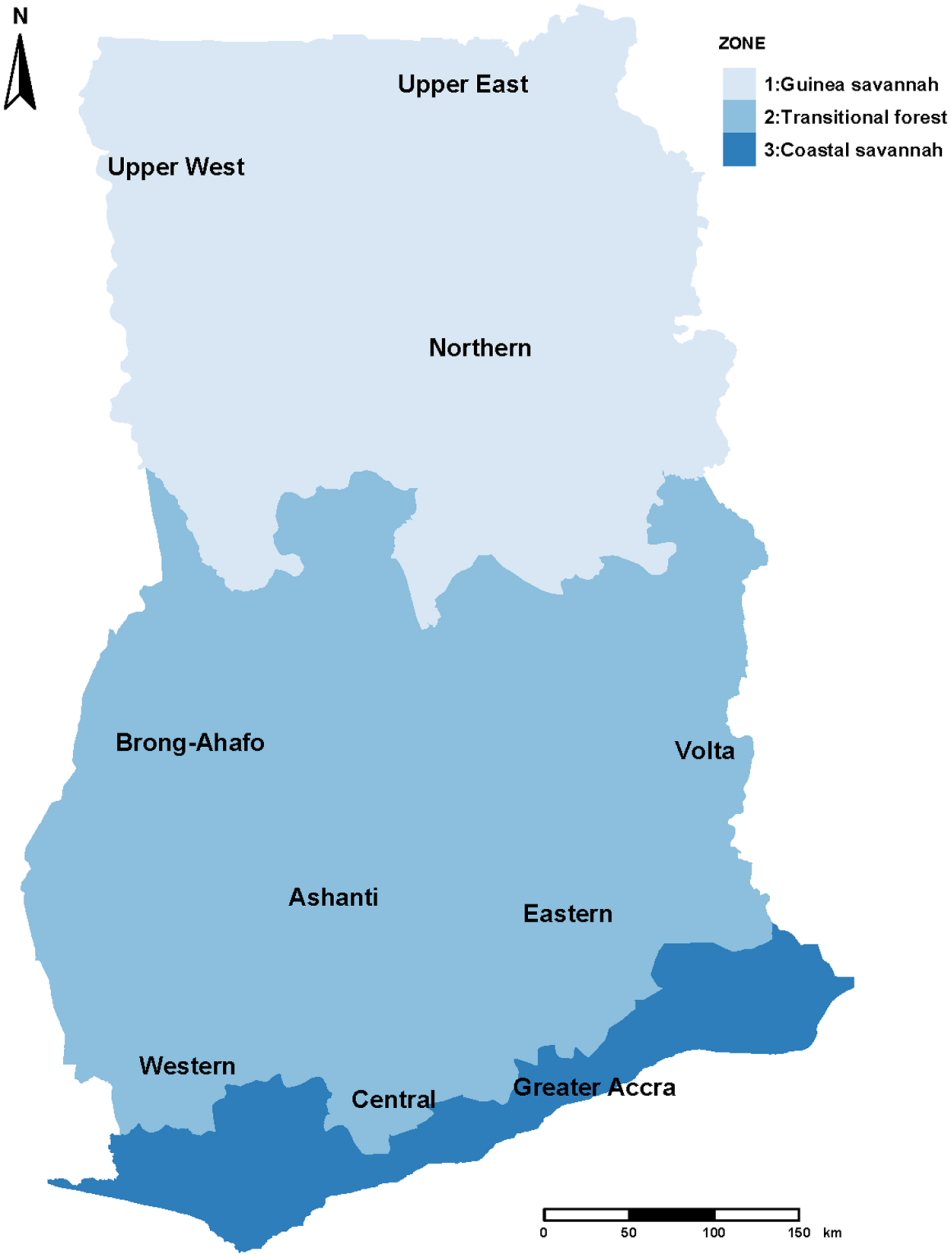
Map of Ghana showing the administrative regions and ecological zones. (**Data source:** https://data.humdata.org/dataset/ghana-administrative-boundaries).

Based on the differences in vegetation, rainfall and temperature patterns recorded over the years, the northern regions (Northern, Upper East and Upper West) of Ghana constitute the Guinea savannah zone while the central regions (Ashanti, Brong-Ahafo, Eastern and mid-Volta) constitute the Transitional forest zone. The Coastal savannah zone includes the Greater Accra, Western and the Central regions. The Coastal savannah also includes the lower Volta boarding the sea, Fig 1.

The Guinea savannah zone experiences a single wet season from June to October and a dry season from November to May. The Transitional forest and Coastal savannah zones experience two wet seasons—major season from April to August and minor season from September to November [9]. The distribution of rainfall in the Transitional forest and Coastal savannah zones are therefore bimodal. The average peak values of rainfall however vary from the Transitional forest to the Coastal savannah zones. The minor wet season peaks higher in the forest zone compared to the coastal zone [9].

## Data

### Clinical data

Each region in the country has a regional hospital that serves as a referral facility. Due to the decentralised nature of health services, lower level health care facilities such as the district hospitals, health centres and clinics are available at the district and sub-district levels and community health facilities such as the Community-based Health Planning and Services (CHPS) exists in the various communities where higher health care facilities are non-existent. These serve as the first points of call for presenting with any illness [1].

In all health facilities (government owned and private), daily morbidity and mortality records are captured and aggregated by week and month into the District Health Information Management System (DHIMS). These include both in and out-patient records of suspected and confirmed malaria cases by the use of microscopy or RDT. Pregnant women attending routine antenatal clinic are also tested for malaria and the results tallied and logged for capture into the DHIMS.

The DHIMS data are anonymised and captured at the district level into a data repository hosted online and made accessible at the regional and national levels. This system of data capture, was set up in 2007 and later revised and upgraded in 2012 [20].

### Meteorological data

Meteorological data from 2008 to 2016 was obtained from the Ghana Meteorological Agency (GMET) in Ghana. Monthly mean minimum and maximum temperature and rainfall from eleven weather stations across all the regions were obtained.

The meteorological data was further aggregated by zone to include the monthly average rainfall in millimeters for all the weather stations within a given zone. Likewise, the monthly mean temperature (minimum and maximum) was determined by month for each zone using recordings from weather stations within their respective zones.

## Methods

Seasonal, trend and residual(from seasonal and trend) components of the malaria case series were investigated using locally estimated scatter plot smoothing (LOESS) decomposition approach [21]. Subsequently, the relationship between confirmed malaria cases and these meteorological factors namely, monthly mean rainfall (mm) and monthly mean temperature (°C) were investigated using time series methods.

To fit autoregressive time series models for the malaria caseloads series, the autocorrelation function (ACF) and partial autocorrelation functions (PACF) of each stationary series were inspected and tentative model parameters determined [22]. Because of the strong seasonal patterns exhibited by all data series for incidence of malaria, rainfall and temperature, Seasonal Autoregressive Integrated Moving Average (SARIMA) models were fitted to each series by zone. Suitable SARIMA models were selected based upon the comparisons of the Akaike Information Criterion (AIC), inspections of the ACF, normal Q-Q plots of residuals and the Box-Ljung test for goodness of fit statistic of all competing models [22].

Determining significant lag terms at which rainfall or temperature that are correlated with cases of malaria after pre-whitening involved inspection of the cross correlation function (CCF) between the residuals of rainfall or temperature and the pre-whitened series of cases of malaria.

Correlation of significant lag terms of rainfall or temperature on cases of malaria (on the negative scale of the CCF as rainfall or temperature leads the incidence of malaria not the other way round) were then extracted from stored values of the CCF.

Parameters of the Impulse response function representing the dynamic relationship between malaria morbidity and meteorological variables were deduced from the cross correlation function of the pre-whitened series of rainfall and temperature. Once the transfer function was determined, an Autoregressive Moving Average (ARMA) model was then determined from the ACF of the transfer function to represent the noise component of the model.

The final transfer function model together with the noise component were then estimated using Ordinary Least Squares algorithm in keeping with the methodology proposed by Box and Jenkins [23]. Associations between malaria morbidity and weather variables at 10% level of significance were considered for inclusion in a multivariable model assessing the combined effect of rainfall and temperature on malaria morbidity. In addition to the usual model diagnostics, predictions from the formulated transfer function models were compared with the observed malaria caseload series.

All data preparation and analyses were performed using Stata version 13.1 (StataCorp LP., College Station, Texas, USA) and R version 3.3.3 Patched Copyright (C) 2017. Analyses were carried out separately for the Guinea savannah, Transitional forest and Coastal savannah zones.

## Results

### Patterns of malaria morbidity, rainfall and temperature

**Fig 2:**
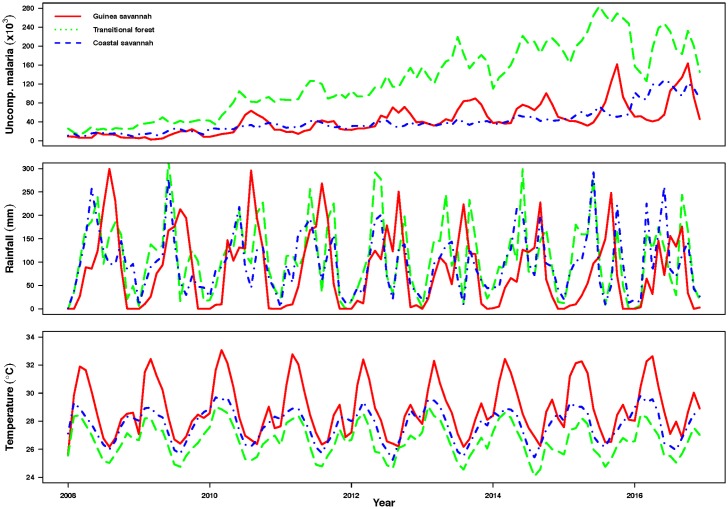
(Top panel) Patterns of uncomplicated malaria morbidity, (Middle panel) average rainfall (mm) and (Lower panel) average temperature (°C) by year for the Guinea savannah, Transitional forest and Coastal savannah zones.

**Fig 3:**
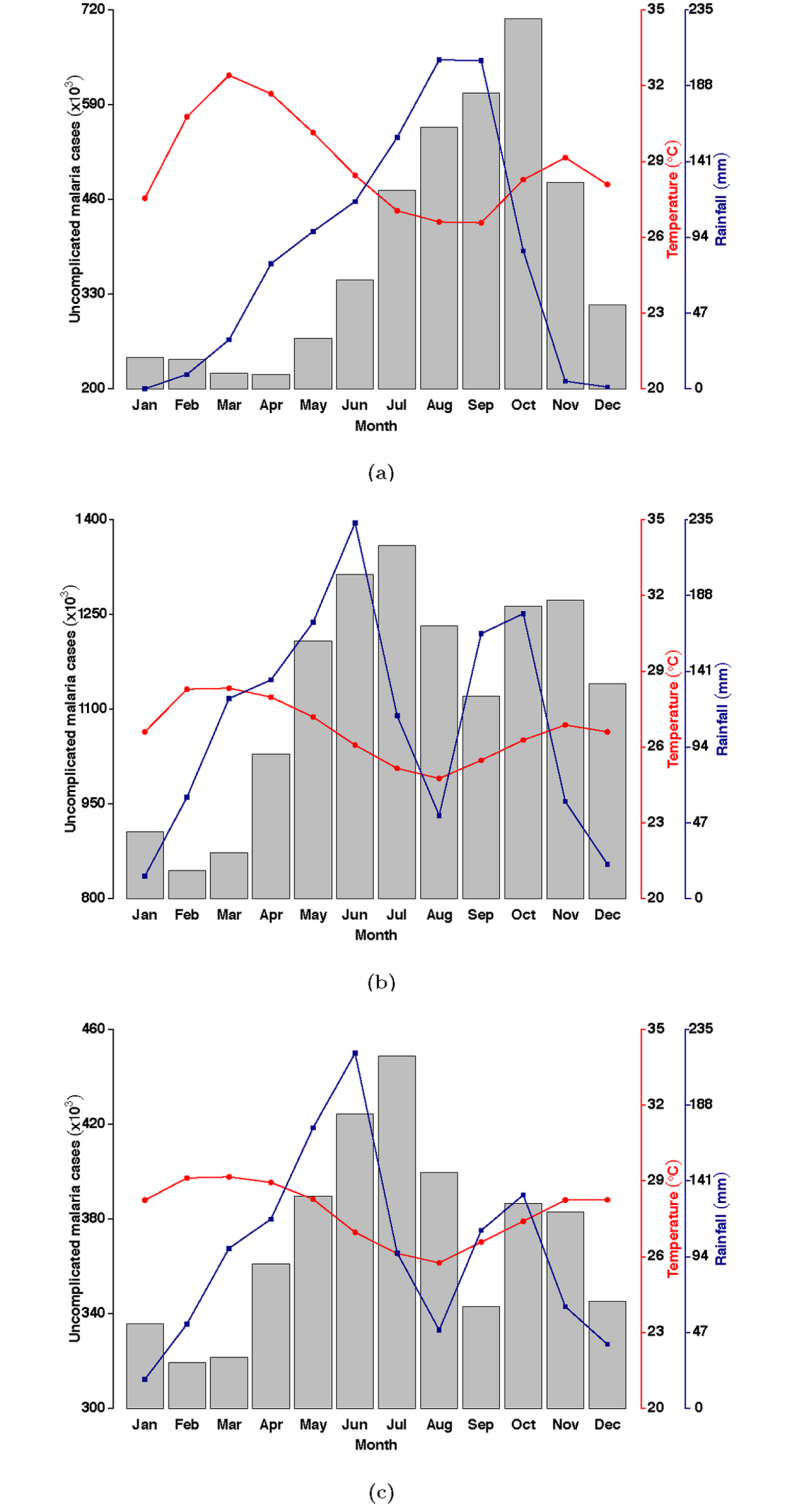
Patterns of aggregated cases of uncomplicated malaria for the (a) Guinea savannah, (b) Transitional forest, (c) Coastal savannah by month for 2008 to 2016.

Figs 2 and 3, shows case series for uncomplicated malaria for the three zones, average rainfall and temperature patterns from 2008 to 2016. Though the seasonal patterns for the malaria data series are not readily obvious in Figs 2 and 3 and S4–S6 Figs show detailed seasonality and a general upward trend from 2008 to 2016 for all zones. Cases of uncomplicated malaria tend to follow an annual pattern with a single peak in the Guinea savannah zone. The peak period of malaria incidence in the savannah coincides with the peak period of the wet season in the zone from May to October, Fig 3a.

From 2008 to 2016, monthly mean number of malaria cases recorded in the Guinea savannah zone was 43,409 with a minimum of 2,471 and maximum of 163,520. Monthly average incidence of uncomplicated malaria in this zone was 915/100,000 (using 2015 estimated population of 4,742,689 from the DHIMS platform). While the monthly mean precipitation in the zone was 82.2 mm with a minimum of 0.0 mm and a maximum of 299.4 mm, average monthly temperature was 28.9°C with the minimum and maximum average temperatures being 25.7°C and 33.1°C for the period respectively (Table 1).

**Table 1:** Summary statistics of data series for Guinea savannah, Transitional forest and Coastal savannah zones.

Summary	Cases of uncomplicated malaria (n)			Average rainfall (mm)			Average temperature (°C)		
	Guinea savannah	Transitional forest	Coastal savannah	Guinea savannah	Transitional forest	Coastal savannah	Guinea savannah	Transitional forest	Coastal savannah
**Minimum**	2471	12124	8185	0.0	0.0	1.3	25.7	24.1	25.3
**Median**	38917	116958	34410	68.1	104.1	90.3	28.5	26.5	28.0
**Mean**	43409	125547	41269	82.2	110.7	97.7	28.9	26.6	27.8
**Maximum**	163520	284428	128228	299.3	316.2	297.4	33.1	29.1	29.8

Relationship between average monthly temperature and incidence of malaria in the Guinea savannah zone on the other hand seems to be in the reverse. While incidence of malaria is rising from May, average temperatures are declining, with the lowest temperature coinciding with the peak of rainfall (Fig 3a).

In the Transitional forest zone, average number of cases of malaria recorded from 2008 to 2016 was 125,547 (Table 1) with average monthly incidence of 796/100,000 people (using 2015 estimated population of 15,762,783 from the DHIMS platform). The lowest and highest average recorded number of malaria cases were 12,124 and 284,428 respectively. Mean, minimum and maximum rainfall recorded within the same period were 110.7 mm, 0.0 mm and 316.2 mm respectively. Also, as shown on Table 1, the mean, minimum and maximum temperatures were 26.6°C, 24.1°C and 29.1°C respectively.

Patterns of uncomplicated malaria in the Transitional forest zone as shown in Fig 3, indicate two distinguishable peaks of malaria incidence mimicking rainfall patterns (middle panel of Fig 2) in the zone. Seasonal patterns extracted after decomposing using LOESS as in S5 Fig also show series for malaria cases shows a double peak.

The rise in number of cases of malaria starts a few months after the onset of the rains, peak in the incidence of malaria follows closely after rainfall peaks, in both the first and second wet seasons (Fig 3b).

Similarly in the Coastal savannah zone, a doubly peaked pattern of malaria incidence was observed (middle panel of Figs 2 and 3c and S6 Fig) with an average of 41,269 cases recorded per month within the period (Table 1). The mean, minimum and maximum rainfall and temperature recorded in the period 2008 to 2016 were 97.7 mm, 1.3 mm, 297.4 mm and 27.8°C, 25.3°C and 29.8°C respectively, Table 1. Average monthly incidence was 553/100,000 people (using 2015 estimated population of 7,466,490 from the DHIMS platform).

Notably, the major peak period for malaria incidence in the Transitional forest and the Coastal savannah zones seem to occur in June (Figs 2, 3b and 3c). Additionally, recorded cases of malaria in the Coastal savannah zone follow similar patterns as in the Transitional zone although the number of cases during the second season in the Coastal savannah zone is relatively lower (Figs 2, 3b and 3c). This difference may be due to the amount of rainfall received and level of temperatures recorded in the respective zones (Figs 2 and 3). Another distinction in the two zones is that, recorded temperatures are slightly higher in the Coastal savannah zone compared to the Transitional forest zone (Figs 2 and 3).

In all three zones, the highest rainfall and the lowest temperatures were recorded in the Transitional forest zone (Figs 2 and 3). In contrast, the Guinea savannah zone recorded the least amount of total rainfall, has the shortest wet season, and seems to be relatively hotter on the average (Figs 2 and 3a).

Since these weather indicators vary across zones, it may be more informative to investigate these relationships on zonal basis in order to better understand the level of dependencies between incidence of malaria, rainfall and temperature.

S1–S3 Figs also show patterns of confirmed severe malaria cases, diagnosed malaria among pregnant women and malaria attributable deaths in all zones. These categories of diagnosed malaria cases, largely follow similar patterns as for the relationship between uncomplicated malaria cases, rainfall and temperature in the various zones although with relatively fewer cases as shown.

### Autoregressive Integrated Moving Average models

The malaria caseload, as well as rainfall and temperature data series from the Guinea savannah were found to have stable variance after testing for unit roots using the Augmented Dicker-Fuller test. A univariate model, SARIMA (2, 0, 1) (1, 0, 0)_12_, was selected for the malaria cases after inspecting the ACF and PACF. Model selection was based on the lowest AIC reported through a grid search by the Auto ARIMA algorithm from the forest package. Similar models were fitted to the malaria caseloads data series in the Transitional forest and Coastal savannah zones. However, the morbidity series from these zones required non-seasonally differencing to stabilize the variance whereas variance stabilisation was not required for rainfall and temperature data. In the Transitional forest zone, SARIMA (1,1,1)(0,0,2)_12_, was the best model with the lowest AIC for malaria morbidity whiles SARIMA (0,1,2)(0,0,1)_12_ was selected for malaria caseloads for the Coastal savannah zone.

These models though limited in application in terms of their usage for making inputs to planning and investigating the impact of malaria interventions, provide some useful insights into the patterns of morbidity of malaria across the country.

### Cross correlation analysis

The correlation coefficients between the raw malaria caseloads, rainfall and temperature in the Guinea savannah zone, without pre-whitening, were 35.0% and 30.0% at lags 1 and 2 with rainfall respectively and -30.0%,-34.0% and -21.0% at lags 0, 1 and 2 with temperature respectively. Those for the Transitional forest were 37.0% and 34.0% at lags 0 and 1 with rainfall respectively and 29.0% and 28.0% at lags 2 and 3 with temperature respectively. Similarly for the Coastal savannah, the correlation between rainfall and malaria morbidity was 25.0% and -22.0% at lags 1 and 2 and 20.0% and 25.0% at lags 1 and 2 with temperature respectively.

From the CCF between pre-whitened series of cases of malaria and rainfall and cases of malaria and temperature, it was observed that the only significant correlations remaining between rainfall, temperature and malaria morbidity occurred at a one month lag respectively in the Guinea savannah.

In the Transitional forest zone, the correlation between rainfall and cases of malaria, after pre-whitening that remained significant were those at lag zero and at one month. With regards to temperature, significant correlations were observed at a two month lag. Similarly for the Coastal savannah zone, remaining significant cross correlations observed from the CCF after pre-whitening malaria caseloads versus rainfall and temperature were at lags one and two months for rainfall and at two months lag with temperature respectively.

In all the zones, increasing rainfall and lowering temperatures preceded the onset of the rise of malaria incidence and timing of the rise in malaria incidence is well defined following the observations made from these analyses.

In the Guinea savannah as well as in the Coastal savannah zones, rainfall leads incidence of malaria by a month whereas in the forest zone, the lead time between rainfall and incidence of malaria is less than a month perhaps due to persistence in rainfall almost all year round.

### Transfer functions

As shown on S1 and S3 Tables, results of the CCF between malaria morbidity rainfall and temperature separately were used to determine the impulse response function for the relationship between malaria caseloads and weather variability.

Univariate models for cases of malaria and rainfall in the Guinea savannah show morbidity lags rainfall and temperature significantly (at 10% level of significance) by one month, S1 Table. In the multivariable model including both rainfall and temperature, temperature remained significantly associated but not rainfall (Table 2 and S1 Table). The random seasonal and non-seasonal error terms however remained highly significant. This may suggest that, the components of the unobserved factors include both seasonal and non-seasonal variables that are contributing significantly to the variability in malaria morbidity.

**Table 2:** Time series multivariable regression estimates of the relationship between average monthly rainfall, temperature and cases of malaria confirmed by zone.

Variables	Guinea Savannah		Transitional Forest		Coastal Savannah	
	Coefficients (95% CI)	p-value	Coefficients (95% CI)	p-value	Coefficients (95% CI)	p-value
Rainfall						
Lag0*	-	-	-	-	-	-
Lag1*	26.77 (-18.75,72.28)	0.249	66.90 (15.48,118.34)	0.011	35.12 (1.06,69.19)	0.043
Lag2*	-	-	-	-	-37.16 (-68.93,-5.40)	0.022
Temperature						
Lag0*	-	-	-	-	-	-
Lag1*	-3962.43 (-8146.65,231.79)	0.064	-	-	-	-
Lag2*	-	-	5198.12 (1882.49,8513.75)	0.002	1143.47 (-206.97,2493.92)	0.097
ARMA**						
AR(1)	0.82 (0.74,0.89)	<0.001	-	-	-0.25 (-0.50,0.002)	0.052
AR(2)	-	-	-0.16 (-0.34,0.02)	0.082	-0.29 (-0.48,-0.10)	0.003
AR(3)	-	-	-0.25 (-0.42,-0.08)	0.004	-	-
SARMA***						
SAR(1)	0.65 (0.45,0.85)	<0.001	0.13 (-0.07, 0.34)	0.199	-	-
Intercept	153855.60 (39883.43,267827.9)	0.008	-144687.40 (-232669.88, -56704.82)	0.001	-30719.38 (-68424.81,6989.05)	0.110
Sigma	11573.52 (10336.88,12810.15)	<0.001	18928.17 (16899.22,20957.13)	<0.001	7355.74 (6692.89,8018.59)	<0.001

* Lag0, Lag1, Lag2: Refer to elapsed times in months (0, 1, 2) for malaria incidence with respect to rainfall and temperature

** ARMA: Autoregressive (AR) and Moving average (MA)

*** SARMA: Seasonal Autoregressive (AR) and Moving average (MA)

In contrast, the univariate models between cases of malaria and rainfall and temperature in the Transitional forest zone show, the delay effect of rainfall on malaria morbidity includes weeks up to a month whereas for temperature the delay is two months. The combined delay effect of rainfall and temperature on malaria morbidity indicates rainfall leads by one month and for temperature the lead is by two months, both being significantly associated with malaria morbidity caseloads (Table 2 and S2 Table).

Even though the patterns of morbidity and meteorological variables look similar comparing the Transitional forest zone and the Coastal savannah zones, the delay effects of rainfall vary slightly. Effect of rainfall in the previous two months significantly influences the incidence of malaria in the Coastal savannah. The effect of temperature in the Coastal savannah is however similar to its observed effect in the Transitional forest zone. These relationships remained significant even in the combined multivariable model of rainfall and temperature and malaria morbidity, Table 2, S2 and S3 Tables.

Notwithstanding the seasonal component of the random error term not being significant, for both model estimates in the Transitional forest and Coastal savannah zones, the non-seasonal random terms were significant. As explained earlier, this may indicate some unobserved non-seasonal factors significantly explain the variability in malaria morbidity apart from temperature and rainfall (Table 2, S2 and S3 Tables).

Transfer function models for the relationship between malaria caseloads as the dependent outcome variable and rainfall and temperature as the independent input variables were developed and represented by the Eqs 1, 2 and 3 below. These are,

$$Cases_{t}=153855.6+26.8B*Rainfall_{t}-3962.4B*Temp_{t}+\frac{1}{\left(1-0.8B\right)\left(1-0.7B^{12}\right)}N_{t}$$ (1)

for the Guinea savannah,

$$\left(1-B\right)Cases_{t}=-144687.4+66.9B*Rainfall_{t}+5198.1B^{2}*Temp_{t}+\frac{1}{\left(1-0.2B^{2}+0.3B^{3}\right)\left(1-0.1B^{12}\right)}N_{t}$$ (2)

for the Transitional forest and

$$\left(1-B\right)Cases_{t}=-30719.4+\left(35.1+37.2B\right)B*Rainfall_{t}+1143.5B^{2}*Temp_{t}+\frac{1}{\left(1+0.3B+0.3B^{2}\right)}N_{t}$$ (3)

for the Coastal savannah zones respectively. Where the backshift operator ***B***^*k*^ = ***X***_*t*−*k*_ and *N*_*t*_ ~ *N*(0,1) i.e. white noise.

**Fig 4:**
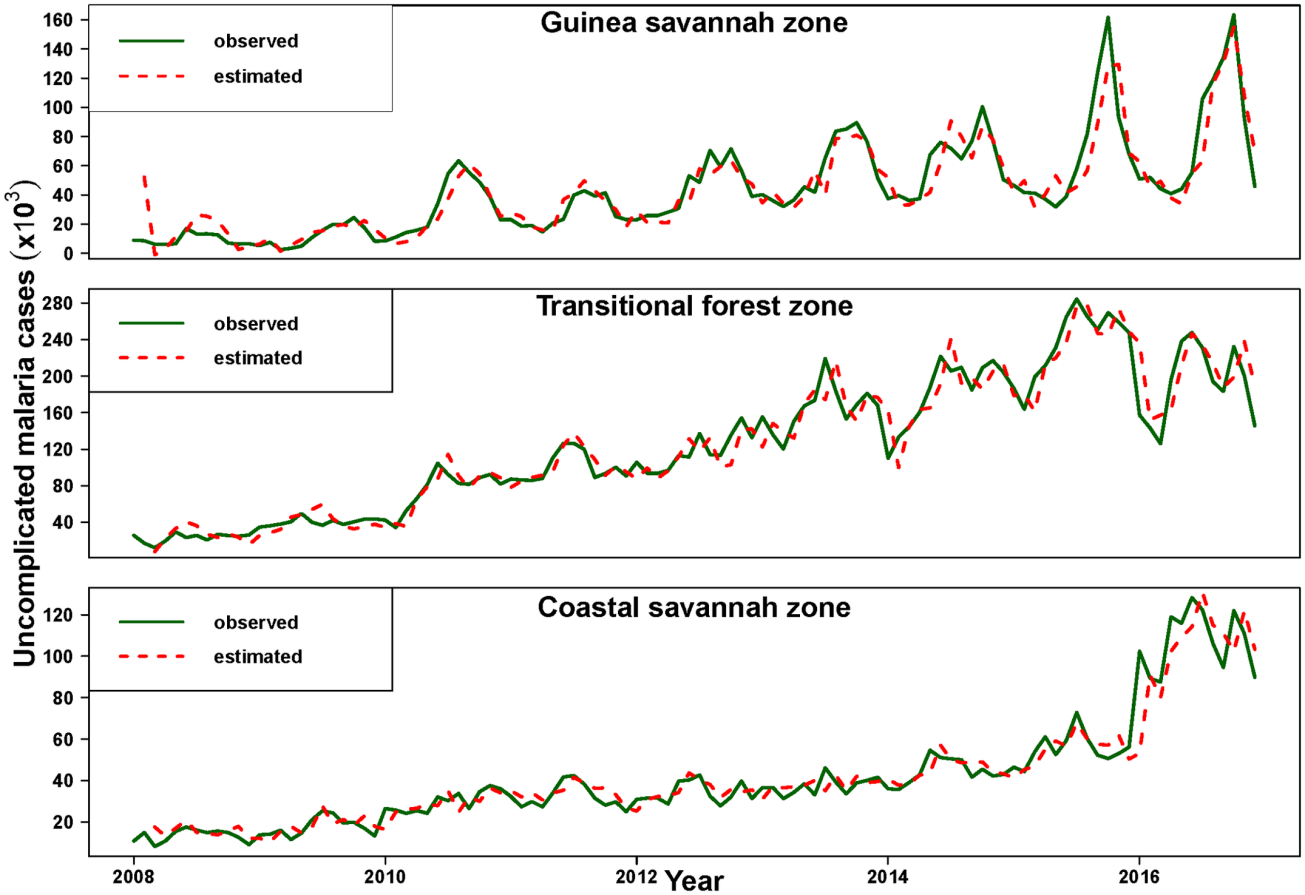
Predicated versus observed uncomplicated malaria cases by zone.

Model predications of malaria morbidity using Eqs (1), (2) and (3), including inspection of the model residuals, Q-Q plots provided assurance of model adequacy. The proportion of variability in the malaria caseloads series accounted for by the fitted models estimated from the coefficient of determination (R-squared) for each model were 86.1%,93.2% and 92.6% respectively for the Guinea savannah, Transitional forest and Coastal savannah models. Fig 4 shows a comparison of the estimated and observed number of cases of malaria by zone.

## Discussion

This study has shown the dynamic features of malaria morbidity using data captured on the DHIMS repository. Notably, the results show distinguishable patterns which correlate with the weather phenomenon in various zones of Ghana. With respect to weather dynamics, these results corroborate some of the findings of some studies investigating variability of meteorological factors across Ghana [9, 16]. Results of spatial and temporal relationships between malaria morbidity and weather variability, using data from the DHIMS platform at the zonal level for the entire country is being reported for the first time, making these findings unique even though a few localised studies have been conducted to investigate the relationship between rainfall and malaria morbidity [15–17].

For example, a study in two districts located in the Transitional forest zone in Ghana found that, the lag time between malaria incidence and rainfall ranged from one to nine weeks [16]. These findings are partially consistent with results of an association of a one month lag with rainfall in the univariable transfer function model for the Transitional zone of this study, S2 Table. The observed inconsistency in findings could have been due to differences in the models fitted. In our study, the unobserved residuals were modelled as autoregressive terms whereas the earlier study [16] in Agogo and Konongo did not model the residual error. Another study investigating the correlations between monthly rainfall and temperature on malaria caseloads observed that, up to two months delay of rainfall was significantly correlated with recorded malaria caseloads in a community (Ejura) located in the Transitional forest zone while mean monthly maximum temperature was correlated with a range of lag time of less than a month to four months in the same community [15]. Similarly, the study found a zero to two month range lag correlation between maximum temperature and malaria caseloads but not rainfall in another community (Winneba) in the Coastal savannah zone [15]. In contrast, results from the Transitional forest zone of our study observed two significant correlations between lag zero and one month with rainfall and malaria incidence and a two months lag of temperature correlation with incidence of malaria respectively. However in the Coastal savannah, the observed significant lagged correlations between incidence of malaria and rainfall were one and two months and two months with temperature respectively. These discrepancies in findings could have resulted from the different analytical approaches adopted, for instance pre-whitening the data series, before assessing the cross correlation in our study. Similarly, another study using district level health facility malaria incidence data from 2008 to 2011 did not find significant associations between incidence of malaria and rainfall but a three month lag correlation with temperature in the Amenfi West district which is situated in the Transitional forest zone [17]. This finding is also not consistent with results of correlations found between the pre-whitened malaria incidence and temperature data series of our study in the Transitional forest zone.

As observed in this study, some significant correlations between the raw data series without pre-whitening disappeared after pre-whitening was applied. This may suggest spurious relationships were observed without pre-whitening as noted by a study conducted in Sri Lanka [24]. Some of the findings in this study were also found to be closely consistent with findings from other studies where less than or up to a month lag in rainfall was found to be related to malaria morbidity caseloads [24, 25].

Though the morbidity data series across all the zones, S4–S6 Figs, seem to suggest an increasing trend (after decomposing the series using LOESS) in malaria morbidity in the country, these observed trends are not necessarily attributable to increasing number of new malaria cases or general worsening of the disease burden. The reasons for the observed increasing trend could be due to the gradual improvement of the DHIMS data capturing platform which was set up in 2007 [1, 20]. Prior to DHIMS and its subsequent upgrading in 2012, the roll out and scaling up of Community-based health planning and services (CHPS) and the systematic increase in the testing rate for malaria diagnoses could have played a significant role in reaching out to a wider population requiring health care who are accurately diagnosed for malaria [26–28]. These trends notwithstanding, the seasonal characteristics of malaria morbidity remain, though with lower records in earlier years as shown in Fig 2 and S4–S6 Figs. The observed increasing trend may be an indicator of a steady increase in the reporting levels of malaria cases across the country which increases the reliability of data from the DHIMS for prediction of the burden of malaria in the country without having to conduct new field studies using limited resources.

The seeming discrepancy between the prevalence and incidence of malaria between the two zones may be explained by the high malaria prevalence in previous years before 2016 in the Guinea savannah zone compared to the other zones. In the 2016 survey among children 6–59 months of age, prevalence of malaria parasite was estimated to be 20.6% nationwide. The prevalence was however 20.4% in the Guinea savannah and 24.3% in the Transitional forest zone respectively in the same year. Prior to 2016, prevalence among children in the same age group have always been estimated to be higher in the Guinea Savannah compared to other zones. For instance in 2011, the average parasite prevalence in the Guinea savannah was 47.8% compared to 24.6% and 24.2% in the Transitional forest and Coastal savannah zones respectively [29].

Given the high prevalence in the Guinea savannah in previous years, and the higher estimated average incidence of malaria in all age groups from 2008 to 2016, it is not unexpected that, the Guinea savannah seem to have recorded higher incidence rates compared to other zones.

The relationship between malaria morbidity and meteorological factors are complex and though the observed relationship may not be directly causal, these findings with regards to rainfall and temperature are plausible given the length of time it takes for parasite development in the human host until clinical illness is reported [13]. While meteorological factors may not be the only factors responsible for the observed relationships, understanding the impact of other similarly important factors may require a more complex modelling approach. For instance, factors such as availability of health facilities, the health seeking behavior of members of the community, socio-economic levels of households, presence of irrigated farm lands and large community dams and dugouts could influence transmission dynamics and therefore the length of time before clinical cases are seen at the health facilities. Prevalence of malaria in the country is all year round, even in the Guinea savannah zone where the unfavourable weather conditions of the dry season for both mosquitoes and parasite development prevail, the many irrigated and non-irrigated farm lands and dams may be serving as breeding grounds for infectious vectors [14, 19]. These factors and many others, such as immigration from higher into low endemic areas, could form part of the unobserved variables accounting for the significance of the noise component in the models.

With this background, the findings here could serve as a guide planning for appropriate interventions to avert seasonal epidemics particularly where seasonality of morbidity has been shown to be significant. This could be done by implementing interventions that target breeding sites in the dry season to reduce or clear the vector population and the parasite reservoir in the human host by mass drug administration, for example and if possible, before the onset of the rainy season [30].

While transfer function models may be better than the univariate models in terms of being able to incorporate a couple of dependencies for predictions of morbidity, they may not be flexible enough to accommodate more complex underlying mechanisms of the disease. Nevertheless, the impulse response functions maybe supportive in developing more elaborate mathematical models.

Going forward, formulating a dynamic mathematical model not only for predictions of disease incidence but also for investigating the impact of malaria interventions such as indoor residual spraying, potential new malaria vaccines or drugs such as the RTSS [31] or a potential single dose antimalarial drug such as the MMV390048 [32] on the incidence of malaria morbidity in each zone, may be supportive in providing the needed information for planning, cost effectiveness analysis and target setting. This will support in addressing some of the challenges in malaria control in Ghana highlighted by the NMCP [33].

These models should be able to take into account the coverage levels of the interventions currently being deployed as well as the health seeking behaviour, the density of functioning health infrastructure in various districts and other factors that may relate to malaria incidence in Ghana while also accounting for the varying patterns of morbidity.

## Conclusion

The data captured on the DHIMS from 2008 to 2016 has been used to demonstrate spatial and temporal heterogeneity of malaria morbidity across ecological zones in Ghana. Additionally, relationships between malaria morbidity and rainfall and malaria morbidity temperature together with the combined effects of both weather variables in the three different zones were explored. Particularly those of the Guinea savannah have been investigated for the first time and the dynamical models and other analytical approaches used seem appropriate and the results obtained robust. Timing of these variations could guide deployment of interventions and treatment strategies such as indoor residual spraying and Seasonal Malaria Chemoprevention or vaccines to optimise effectiveness. A more elaborate modelling approach that allows for more dependencies of malaria morbidity dynamics to be incorporated may provide a platform to investigate the impact of such intervention strategies using this data. It is important to note, that the increasing trends of captured morbidity cases at the health facilities seems to indicator that, some positive achievements have been made in recent years in improving both health care coverage levels and data reliability.

## Ethical considerations

Ethical approval for this study was obtained from the Institutional Review Board of the Navrongo Health Research Centre, Ghana and the University Of Cape Town Faculty Of Science Research Ethics Committee. Access and Permission to use malaria data captured on the DHIMS was granted by the National Malaria Control Program, Ghana.

## Supporting information

S1 Table Time series regression estimates of the relationship between average monthly rainfall, temperature and cases of malaria confirmed in the Guinea savannah zone. (DOCX)

S2 Table Time series regression estimates of the relationship between average monthly rainfall, temperature and cases of malaria confirmed in the Transitional forest zone. (DOCX)

S3 Table Time series regression estimates of the relationship between average monthly rainfall, temperature and cases of malaria confirmed in the Coastal savannah zone. (DOCX)

S1 Fig Patterns of aggregated cases of severe malaria for the (a) Guinea savannah, (b) Transitional forest, (c) Coastal savannah by month for 2008 to 2015. (TIF)

S2 Fig Patterns of aggregated cases of malaria in pregnancy for the (a) Guinea savannah, (b) Transitional forest, (c) Coastal savannah by month for 2008 to 2016. (TIF)

S3 Fig Patterns of aggregated cases of malaria attributable deaths for the (a) Guinea savannah, (b) Transitional forest, (c) Coastal savannah by month for 2008 to 2015. (TIF)

S4 Fig Seasonal, trend and remainder (residuals from both season and trend) of the decomposed uncomplicated malaria data series by LOESS for the Guinea savannah zone. (TIF)

S5 Fig Seasonal, trend and remainder (residuals from both season and trend) of the decomposed uncomplicated malaria data series by LOESS for the Transitional forest zone. (TIF)

S6 Fig Seasonal, trend and remainder (residuals from both season and trend) of the decomposed uncomplicated malaria data series by LOESS for the Coastal savannah zone. (TIF)

S1 Dataset Minimal clinical dataset of uncomplicated malaria cases by year, month and zone. (XLSX)
