# Supplementary material for: Spatio-temporal heterogeneity of malaria morbidity in Ghana: Analysis of routine health facility data
Source: PLoS One. 2018 Jan 29;13(1):e0191707. doi: 10.1371/journal.pone.0191707 (PMC5788359; doi:10.1371/journal.pone.0191707)
Supplement: S1 Table — (DOCX) [file pone.0191707.s001.docx]

**S1 Table. Time series regression estimates of the relationship between average monthly rainfall, temperature and cases of malaria confirmed in the Guinea savannah zone.**

| Variables | **Univariate models** | | | | **Multivariable model** | |
| --- | --- | --- | --- | --- | --- | --- |
|  | Rainfall | | Temperature | | Rainfall and Temperature | |
|  | Coefficients (95% CI) | p-value | Coefficients (95% CI) | p-value | Coefficients (95% CI) | p-value |
| Rainfall |  |  |  |  |  |  |
| Lag0^*^ | - |  | - |  | - | - |
| Lag1^*^ | 39.80 (-3.55,83.10) | 0.072 | - |  | 26.77 (-18.75,72.28) | 0.249 |
| Lag2^*^ | - |  | - |  | - | - |
| Temperature |  |  |  |  |  |  |
| Lag0^*^ | - |  | - |  | - | - |
| Lag1^*^ | - |  | -4017.40 (-8004.60,-30.30) | 0.048 | -3962.43 (-8146.65,231.79) | 0.064 |
| Lag2^*^ | - |  | - |  | - |  |
| ARMA^**^ |  |  |  |  |  |  |
| AR(1) | 0.80 (0.71,0.89) | <0.001 | 0.95 ((0.81,1.10) | <0.001 | 0.82 (0.74,0.89) | <0.001 |
| AR(2) | - |  | -0.16 (-0.35,-0.03) | 0.093 | - | - |
| SARMA^***^ |  |  |  |  |  |  |
| SAR(1) | 0.70 (0.53,0.87) | <0.001 | 0.66 (0.47,0.84) | <0.001 | 0.65 (0.45,0.85) | <0.001 |
| Intercept | 38938.38 1660.45,76216.31) | 0.041 | 157890.9 (49299.0,266482.8) | 0.004 | 153855.60 (39883.43,267827.9) | 0.008 |
| Sigma | 11849.26 (10585.93,13112.59) | <0.001 | 11504.0 (10276.1,12732.0) | <0.001 | 11573.52 10336.88,12810.15) | <0.001 |

^*^Lag0, Lag1, Lag2: Refer to elapsed times in months (0, 1, 2) for malaria incidence with respect to rainfall and temperature

^**^ ARMA: Autoregressive (AR) and Moving average (MA)

^***^ SARMA: Seasonal Autoregressive (AR) and Moving average (MA)
