# Supplementary material for: Spatio-temporal heterogeneity of malaria morbidity in Ghana: Analysis of routine health facility data
Source: PLoS One. 2018 Jan 29;13(1):e0191707. doi: 10.1371/journal.pone.0191707 (PMC5788359; doi:10.1371/journal.pone.0191707)
Supplement: S2 Table — (DOCX) [file pone.0191707.s002.docx]

**S2 Table. Time series regression estimates of the relationship between average monthly rainfall, temperature and cases of malaria confirmed in the Transitional forest zone.**

| Variables | **Univariate models** | | | | | **Multivariable model** | |
| --- | --- | --- | --- | --- | --- | --- | --- |
|  | Rainfall | | | Temperature | | Rainfall and Temperature | |
|  | Coefficients (95% CI) | p-value | | Coefficients (95% CI) | p-value | Coefficients (95% CI) | p-value |
| Rainfall |  |  |  | |  |  |  |
| Lag0^*^ | 74.15 (15.41,132.90) | 0.013 | - | |  | - | - |
| Lag1^*^ | 63.49 (9.83,117.15) | 0.020 | - | |  | 66.90 (15.48,118.34) | 0.011 |
| Lag2^*^ | - |  | - | |  | - | - |
| Temperature |  |  |  | |  |  |  |
| Lag0^*^ | - |  | - | |  | - | - |
| Lag1^*^ | - |  | - | |  | - | - |
| Lag2^*^ | - |  | 5412.48 (1674.67,9150.29) | | 0.005 | 5198.12 (1882.49,8513.75) | 0.002 |
| ARMA^**^ |  |  |  | |  |  |  |
| AR(1) | -0.03(-0.21,0.14) | 0.713 | - | |  | - | - |
| AR(2) | - |  | -0.18(-0.33,-0.03) | | 0.021 | -0.16(-0.34,0.02) | 0.082 |
| AR(3) | - |  | -0.24(-0.41,-0.07) | | 0.005 | -0.25(-0.42,-0.08) | 0.004 |
| SARMA^***^ |  |  |  | |  |  |  |
| SAR(1) | 0.20 (-0.03,0.43) | 0.083 | 0.23 (0.02, 0.44) | | 0.032 | 0.13 (-0.07, 0.34) | 0.199 |
| Intercept | -14471.84 (-23300.96,-5642.72) | 0.001 | -142996.8 (-242308.50,-43685.11) | | 0.005 | -144687.40 (-232669.88, -56704.82) | 0.001 |
| Sigma | 19393.94 (17506.23,21281.66) | <0.001 | 19762.7 (17834.2,21691.2) | | <0.001 | 18928.17 (16899.22,20957.13) | <0.001 |

^*^Lag0, Lag1, Lag2: Refer to elapsed times in months (0, 1, 2) for malaria incidence with respect to rainfall and temperature

^**^ARMA: Autoregressive (AR) and Moving average (MA)

^***^ SARMA Seasonal: Autoregressive (AR) and Moving average (MA)
