# Supplementary material for: Spatio-temporal heterogeneity of malaria morbidity in Ghana: Analysis of routine health facility data
Source: PLoS One. 2018 Jan 29;13(1):e0191707. doi: 10.1371/journal.pone.0191707 (PMC5788359; doi:10.1371/journal.pone.0191707)
Supplement: S3 Table — (DOCX) [file pone.0191707.s003.docx]

**S3 Table. Time series regression estimates of the relationship between average monthly rainfall, temperature and cases of malaria confirmed in the Coastal savannah zone.**

| Variables | **Univariate models** | | | | **Multivariable model** | |
| --- | --- | --- | --- | --- | --- | --- |
|  | Rainfall | | Temperature | | Rainfall and Temperature | |
|  | Coefficients (95% CI) | p-value | Coefficients (95% CI) | p-value | Coefficients (95% CI) | p-value |
| Rainfall |  |  |  |  |  |  |
| Lag0^*^ | - |  | - |  | - | - |
| Lag1^*^ | 44.92 (11.92,77.9) | 0.008 | - |  | 35.12 (1.06,69.19) | 0.043 |
| Lag2^*^ | -49.52 (-82.07,-16.96) | 0.003 | - |  | -37.16 (-68.93,-5.40) | 0.022 |
| Temperature |  |  |  |  |  |  |
| Lag0^*^ | - | - | - |  | - | - |
| Lag1^*^ | - | - | - |  | - | - |
| Lag2^*^ | - | - | 1784.35 (561.35,3007.35) | 0.004 | 1143.47 (-206.97,2493.92) | 0.097 |
| ARMA^**^ |  |  |  |  |  |  |
| AR(1) | -0.21 (-0.42,0.01) | 0.061 | -0.29 (-0.56, -0.02) | 0.037 | -0.25 (-0.50,0.002) | 0.052 |
| AR(2) | -0.32 (-0.50, -0.13) | 0.001 | -0.34 (-0.48,-0.19) | <0.001 | -0.29 (-0.48,-0.10) | 0.003 |
| SARMA^***^ |  |  |  |  |  |  |
| SAR(1) | 0.28 (-0.12, 0.67) | 0.166 | 0.20 (-0.23,0.63) | 0.363 | - | - |
| Intercept | 1354.44 (-3425.65,6134.53) | 0.579 | -48620.98 (-82638.53,-14603.43) | 0.005 | -30719.38 (-68424.81,6989.05) | 0.110 |
| Sigma | 7395.76 (6682.38,8109.15) | <0.001 | 7620.85 (7056.99,8184.71) | <0.001 | 7355.74 (6692.89,8018.59) | <0.001 |

^*^ Lag0, Lag1, Lag2: Refer to elapsed times in months (0, 1, 2) for malaria incidence with respect to rainfall and temperature

^**^ARMA: Autoregressive (AR) and Moving average (MA)

^***^SARMA^:^ Seasonal Autoregressive (AR) and Moving average (MA)
