# Supplementary figures and images for: Spatio-temporal heterogeneity of malaria morbidity in Ghana: Analysis of routine health facility data
Source: PLoS One. 2018 Jan 29;13(1):e0191707. doi: 10.1371/journal.pone.0191707 (PMC5788359; doi:10.1371/journal.pone.0191707)

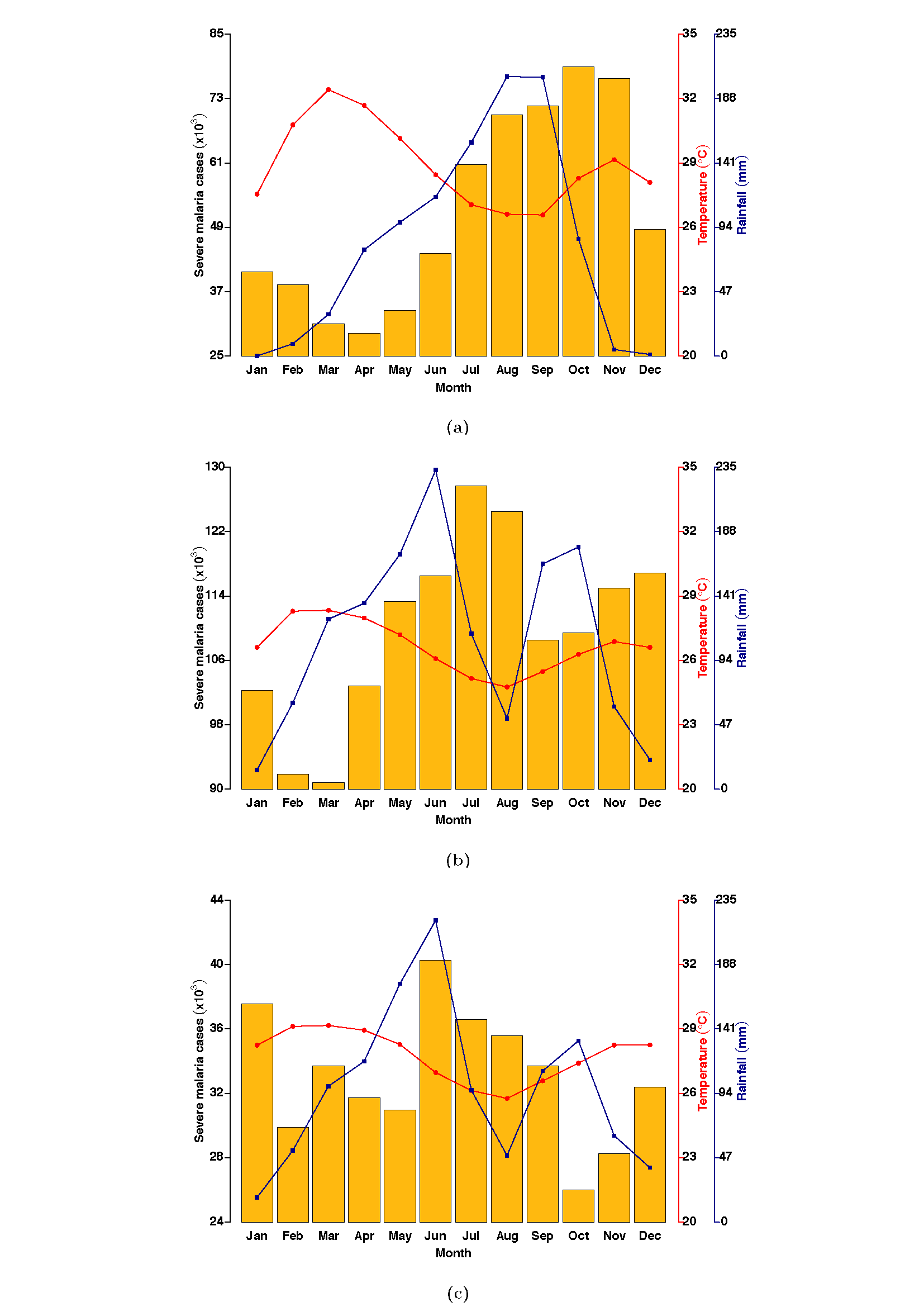

Supplement: S1 Fig — (TIF) [file pone.0191707.s004.tif]

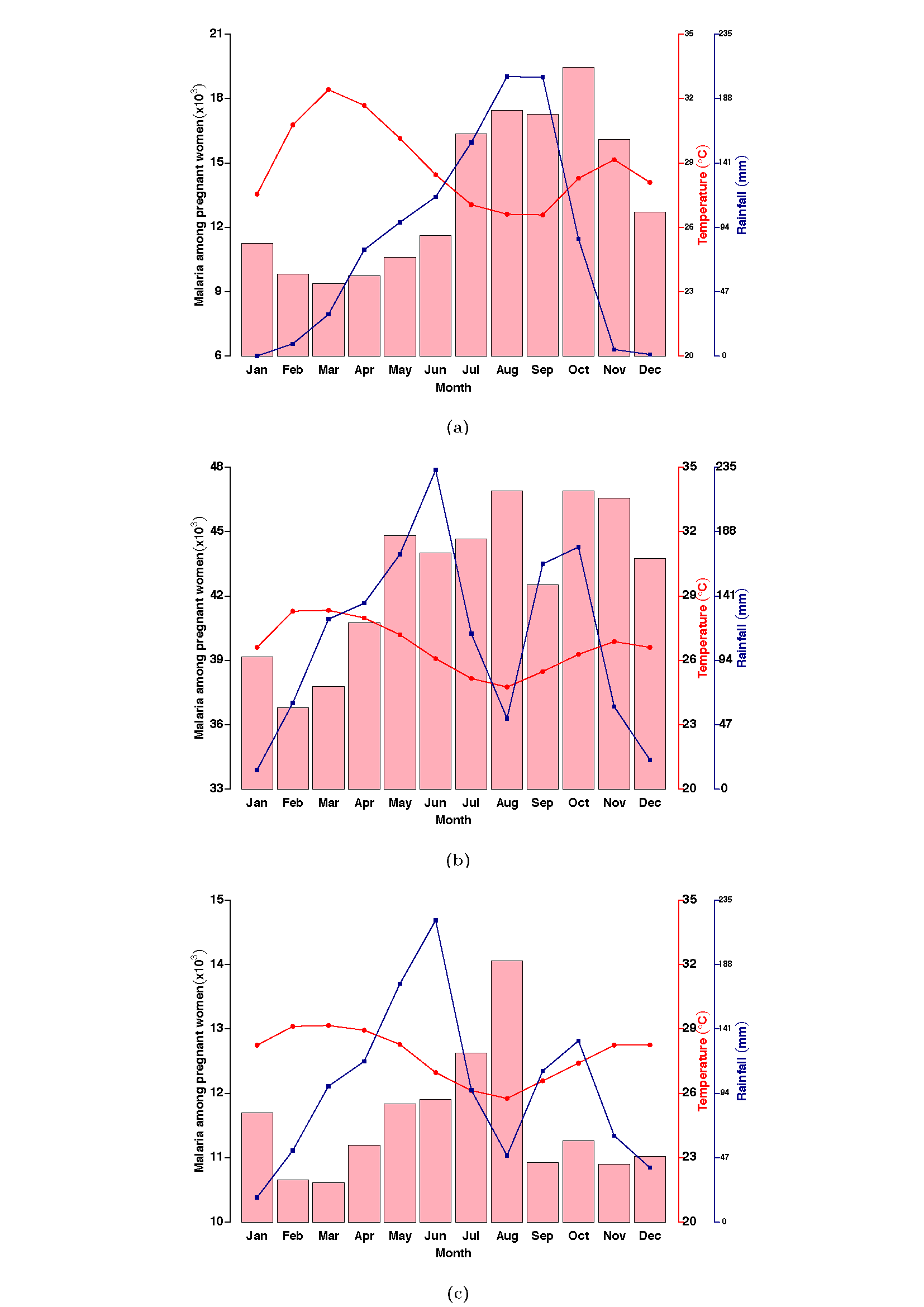

Supplement: S2 Fig — (TIF) [file pone.0191707.s005.tif]

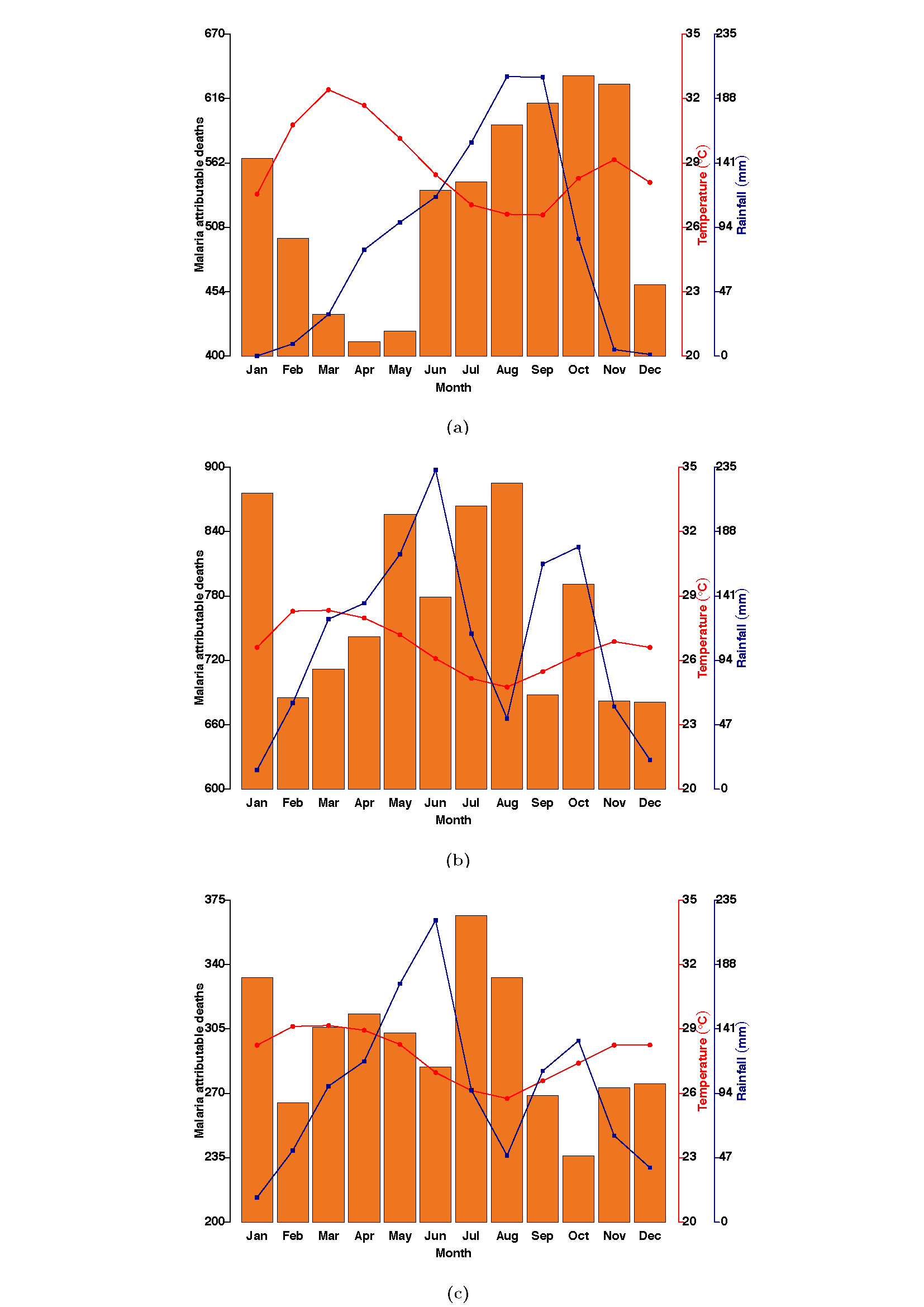

Supplement: S3 Fig — (TIF) [file pone.0191707.s006.tif]

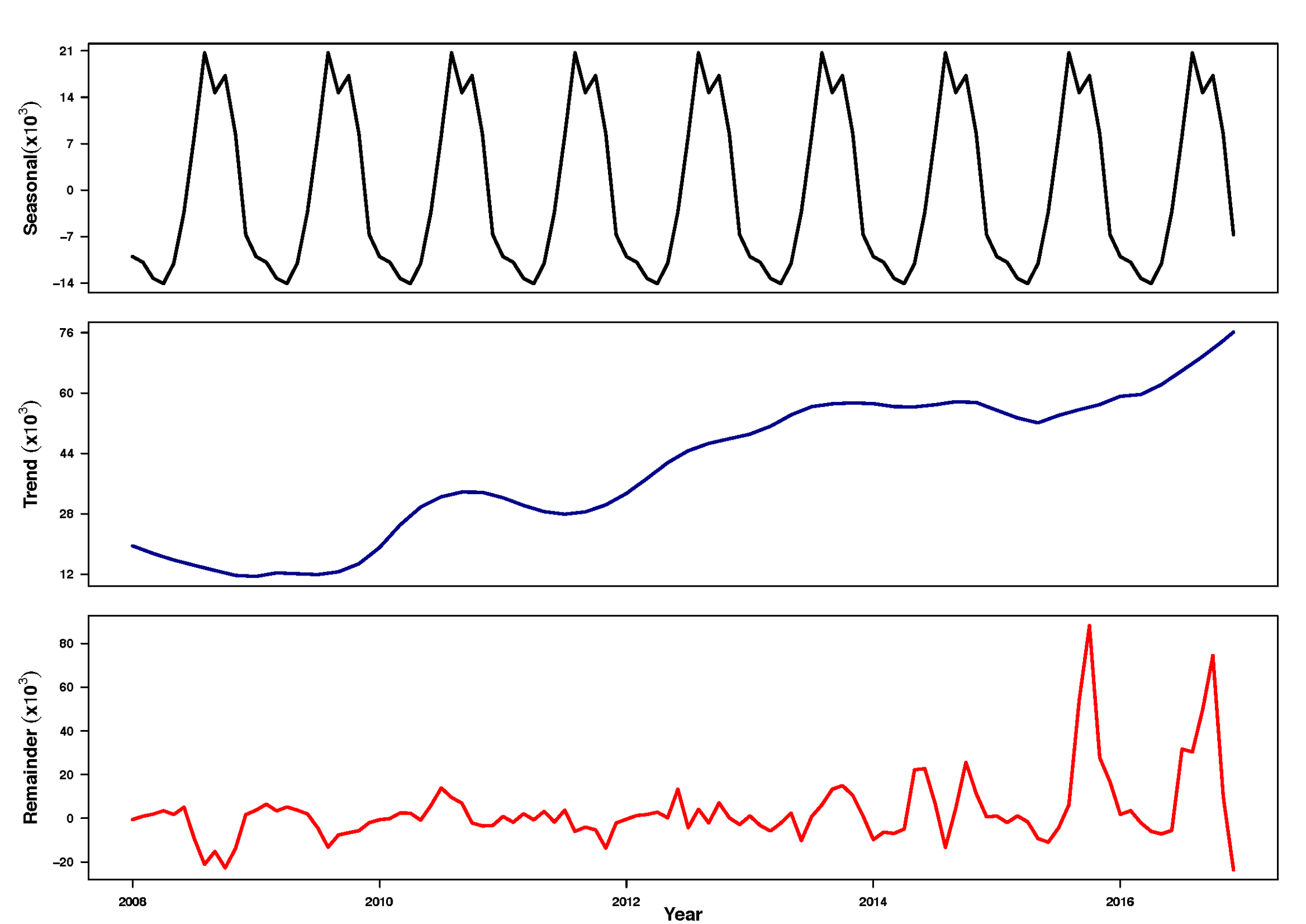

Supplement: S4 Fig — (TIF) [file pone.0191707.s007.tif]

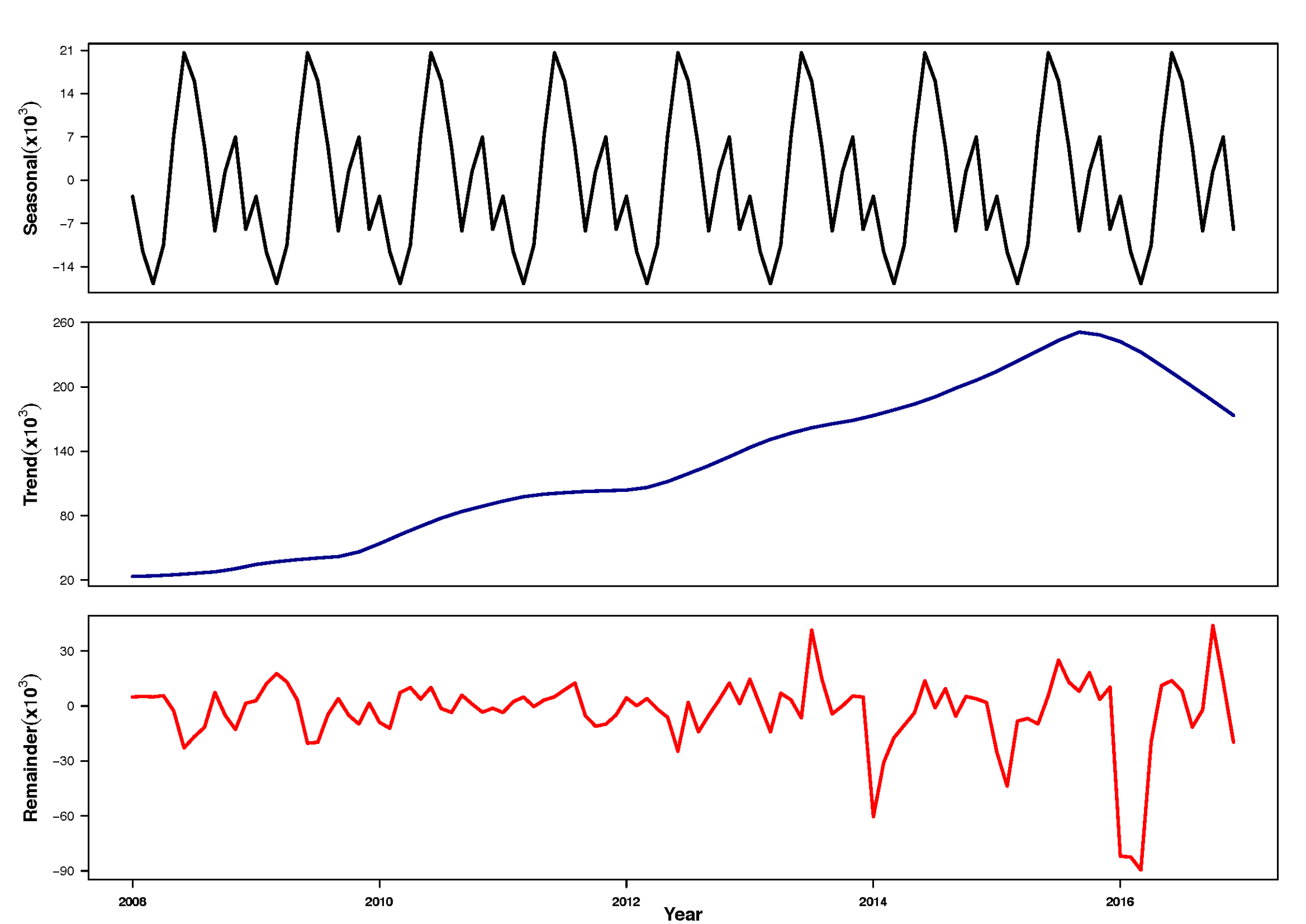

Supplement: S5 Fig — (TIF) [file pone.0191707.s008.tif]

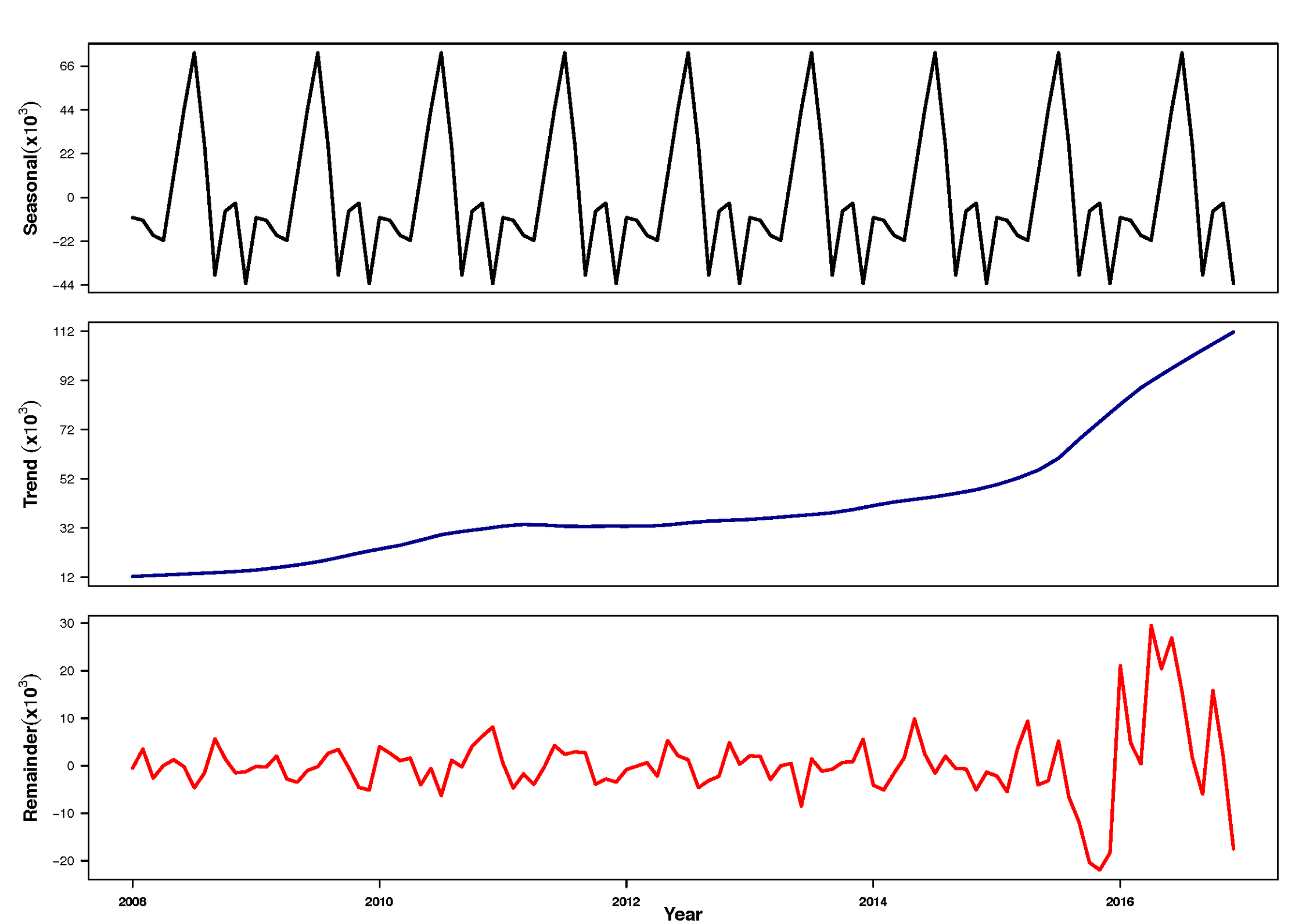

Supplement: S6 Fig — (TIF) [file pone.0191707.s009.tif]
